# Assessment of the Susceptibility of Clinical Gram-Negative and Gram-Positive Bacterial Strains to Fosfomycin and Significance of This Antibiotic in Infection Treatment

**DOI:** 10.3390/pathogens11121441

**Published:** 2022-11-30

**Authors:** Beata Kowalska-Krochmal, Beata Mączyńska, Danuta Rurańska-Smutnicka, Anna Secewicz, Grzegorz Krochmal, Małgorzata Bartelak, Aleksandra Górzyńska, Klaudyna Laufer, Krystyna Woronowicz, Joanna Łubniewska, Jolanta Łappo, Magdalena Czwartos, Ruth Dudek-Wicher

**Affiliations:** 1 Department of Pharmaceutical Microbiology and Parasitology, Faculty of Pharmacy, Wroclaw Medical University, Borowska 211, 50-556 Wroclaw, Poland; 2 Department of Laboratory Diagnostics, Jan Mikulicz-Radecki University Teaching Hospital, Borowska 213, 50-556 Wrocław, Poland; 3 Synevo, T. Marciniak Lower Silesian Specialized Hospital, Gen. A. E. Fieldorfa 2, 54-049 Wrocław, Poland; 4 Microbiological Laboratory, Provincial Specialized Hospital in Legnica, Iwaszkiewicza 5, 59-220 Legnica, Poland; 5 Microbiological Laboratory, A. Sokolowski Specialized Hospital in Walbrzych, Health Department in Klodzko, A. Sokołowskiego 4, 58-309 Walbrzych, Poland; 6 Bacteriology Laboratory, Provincial Specialized Hospital in Klodzko, Szpitalna 1A, 57-300 Klodzko, Poland

**Keywords:** IV fosfomycin, in vitro susceptibility, agar dilution method, Gram-positive and Gram-negative strains, multidrug-resistant strains

## Abstract

Multidrug resistance of bacteria has prompted intensive development work on new medicines, but also the search for effective options among the oldest antibiotics. Although intravenous fosfomycin (IVFOS) seems to be an interesting proposal, the recommended agar dilution method for susceptibility determination poses a major problem in routine diagnostic testing. As a consequence, there is a lack of comprehensive data on the frequency of isolation of susceptible or resistant strains. This fact triggered the disposition of EUCAST concerning the revision of IVFOS breakpoints (BPs), including withdrawal of BPs for *Enterobacterales* (excluding *E. coli*) and coagulase-negative staphylococci. Therefore, the aim of this study was to assess the activity of fosfomycin against numerous clinical strains using recommended methods. **Materials and methods**: A total of 997 bacterial strains were tested from the following genera: *Enterobacterales*, *Pseudomonas* spp., *Staphylococcus* spp., *Acinetobacter* spp., and *Enterococcus* spp., for which there are currently no BPs. The strains were isolated from various clinical materials from patients hospitalized in five hospitals. During the investigation, the recommended agar dilution method was used. Susceptibility to other antibiotics and resistance mechanisms were determined using an automatic method (Phoenix) the disk diffusion method, and E-tests. MIC values of fosfomycin were estimated for all strains and for susceptible and multidrug-resistant (MDR) strains individually. **Results**: Except for *Acinetobacter* and *Enterococcus*, 83% of the strains were susceptible to IVFOS, including the largest percentage of *S. aureus* and *E. coli*. *Klebsiella* spp. turned out to be the least susceptible strains (66%). The highest proportion of susceptibility to fosfomycin was found among strains that were sensitive to other antibiotics (80.9%), and the lowest was found among Gram-negative carbapenemase-producing bacteria (55.6%) and ESBL+ bacteria (61.6%). The MIC evaluation revealed the lowest MIC_50_ and MIC_90_ values for *S. aureus* (0.5 mg/L and 1 mg/L, respectively) and *E. coli* (4 mg/L and 32 mg/L, respectively). The highest values of MIC_50_ were found for *Acinetobacter* spp. (256 mg/L), while the highest values of MIC_90_ were found for *Acinetobacter* spp. and *Klebsiella* spp. (256 mg/L and 512 mg/L, respectively). **Conclusions**: IVFOS appears to be suitable for the treatment of many infections, including the empirical treatment of polymicrobial infections and those caused by MDR strains, since the sensitivity of the studied strains to this antibiotic in different groups ranged from 66% to as much as 99%. Sensitivity to fosfomycin was also demonstrated by 60% of carbapenem-resistant strains; therefore, IVFOS is one of the few therapeutic options that can be effective against the most resistant Gram-negative rods. In light of the general consultation posted by EUCAST, obtaining data such as IVFOS MIC value distributions may be vital for the decision of implementing fosfomycin into breakpoint tables.

## 1. Introduction

Multidrug resistance of bacterial strains has triggered intensive development work on new antibiotics as well as the search for effective options among the oldest ones. There has been a renewed interest in colistin, despite its imperfect pharmacokinetic profile. The above is a result of outcomes of microbiological tests that often indicate colistin as the only therapeutic option against Gram-negative rods. In recent years, fosfomycin has also been identified as an antibiotic with potential activity against multidrug-resistant (MDR) bacteria. In many countries, an oral form of fosfomycin (fosfomycin tromethamine) has been used for a long time, but the application of this form is limited to the treatment of uncomplicated urinary tract infections (UTIs). Recently, however, attention has been drawn to its significance in the treatment of bacterial prostatitis—especially its chronic form. The oral form of fosfomycin may be used as an alternative treatment option to fluoroquinolones in UTIs, including complicated cases [1,2]. On the other hand, the intravenous form of fosfomycin (fosfomycin disodium; IVFOS) has much wider indications than its oral form. Therefore, IVFOS is currently drawing attention worldwide. According to the European Medicines Agency Assessment Report dated 26 March 2020, IV fosfomycin may be used in the treatment of methicillin-resistant staphylococcal meningitis, encephalitis, abscess of the brain, osteomyelitis, complicated urinary tract infections, nosocomial lower respiratory tract infections, skin and soft tissue infections, burn infections, biliary tract infections, oto-, rhino-, and laryngological infections, ophthalmological infections, endocarditis, and bacteremia that occurs in association with or is suspected to be associated with the infections listed above. First of all, however, IVFOS is recommended in severe infections caused by fosfomycin-susceptible Gram-negative pathogens with limited therapeutic options [3]. Fosfomycin’s ability to penetrate many organs is, among others, a result of the small size of its molecules. Fosfomycin has the smallest molecular mass among all known antibacterial drugs [4,5,6]. Its mechanism of action involves inhibiting the activity of *UDP-N-Acetylglucosamine Enolpyruvyl Transferase* (MurA), which is responsible for the formation of N-acetylmuramic acid—a precursor of peptidoglycan. For its activity, fosfomycin requires the glycerol-3-phosphate (G3P) and glucose-6-phosphate (G6P) transporters. No cross-resistance with other antibiotics has been reported [7,8]. Fosfomycin displays a broad spectrum of activity. It is effective against *Staphylococcus aureus*—including methicillin-resistant *S. aureus* (MRSA)—MDR *E. coli*, and extensively drug-resistant (XDR) strains. The literature also mentions its potential activity against other Gram-positive bacteria (e.g., *Staphylococcus epidermidis*, including methicillin-resistant *S. epidermidis* (MRSE); *Streptococcus pneumoniae*, including penicillin-resistant *S. pneumoniae* (PRSP); *Enterococcus* spp., including vancomycin-resistant enterococci (VRE); and *Enterobacterales* other than *E. coli* and *Pseudomonas aeruginosa*, including MDR and XDR strains) [3,4,7,8,9,10]. According to the latest recommendations, IVFOS should not be used as a monotherapy. One of the reasons is a rapid increase in resistance to this antibiotic caused by various mechanisms. Among them is the production of glutathione S-transferase (FosA) by Gram-negative bacteria, which inactivates fosfomycin [4,11]. The need to use fosfomycin in combination therapy has been stressed for a long time, particularly in the treatment of *P. aeruginosa* infections [10].

The intravenous form is not as widely available as the oral form, and it is still not approved in the United States. In Europe, it is available in countries such as Germany, Greece, France, Spain, the Netherlands, Austria, the United Kingdom and, since August 2019, also in Poland.

At present, the greatest challenge is the assessment of susceptibility to fosfomycin, for which a quantitative agar dilution method is recommended [12]. Because it is labor- and time-consuming, this method is practically never used in laboratories routinely performing microbiological tests. The undoubted advantages of this method, such as simple interpretation and high reproducibility of results [13], are outweighed by a number of drawbacks accompanying its implementation (e.g., possibility of inaccurate drug dilutions, antibiotic inactivation due to high temperature of agar, etc.). The solution could be readily available, easy-to-use, and certified commercial kits (such as AD Fosfomycin 0.25-256 Liofilchem, Waltham, MA, USA), if not for their high price [14]. Therefore, the evaluation of the sensitivity to IVFOS is carried out using automatic systems such as BD Phoenix or by gradient diffusion method E-tests (bioMérieux, M.I.C.E–OXOID) which, according to data from the literature, may lead to false results for fosfomycin. The lack of a simple and reliable method is also the reason behind the lack of cumulative data on the frequency of isolation of strains susceptible or resistant to IVFOS in individual hospitals, countries, or regions of the world, with such reports being crucial for the determination of empirical therapy regimens. This problem is exacerbated by the fact that there are no clear rules for the interpretation of results based on the currently known breakpoints (BPs). In May 2022, EUCAST presented proposals for new IVFOS BPs for discussion (consultation from 14 May to (extension) 15 July 2022). According to the announced proposals, criteria for the interpretation of susceptibility to IVFOS will be available only for *E. coli* and *S. aureus*, because there are insufficient data—including on minimum inhibitory concentration (MIC) values—for such interpretation criteria to be determined for other bacteria [15]. Unfortunately, if the new EUCAST proposals are accepted, laboratories will discontinue assessments of sensitivity to fosfomycin for most bacterial strains, including *Klebsiella pneumoniae* and *P. aeruginosa*. The lack of information on microbiological results may lead to withdrawal of IVFOS from therapy in situations where its use could be clinically beneficial.

With the above in mind, in this study we performed IVFOS susceptibility assessments for 997 different clinical strains using the reference agar dilution method. The tested strains included both sensitive and resistant Gram-positive cocci and Gram-negative rods. In MIC value analysis, susceptibility differences, the type and origin of strains, and resistance mechanisms were taken into consideration.

## 2. Materials and Methods

### 2.1. Bacterial Strains

This study used 997 bacterial strains from the collection of the Department of Pharmaceutical Microbiology and Parasitology, Wroclaw Medical University, Poland. All strains were isolated in 2021 from patients hospitalized in five hospitals in Lower Silesia in Poland: Jan Mikulicz-Radecki University Teaching Hospital (n = 522), T. Marciniak Lower Silesian Specialized Hospital in Wroclaw (n = 235), A. Sokolowski Specialized Hospital in Walbrzych (n = 114), the Provincial Specialized Hospital in Klodzko (n = 67), and the Provincial Specialized Hospital in Legnica (n = 59). Most of the examined strains came from patients hospitalized in the following departments: various surgery departments (n = 232), intensive care units (n = 202), internal medicine (n = 169), neurology (n = 96), nephrology (n = 73), urology (46), and cardiology (39), as well as others from hematology, pediatric wards, otolaryngology, gynecology, gastroenterology, ophthalmology, and hospital outpatient clinics (n = 140). The tested strains included different groups of Gram-negative and Gram-positive microorganisms, such as the following:

- Gram-negative bacilli from the order *Enterobacterales* (n = 546):
  - *Klebsiella* (n = 250);
  - *E. coli* (n = 181);
  - *Enterobacter* (n = 47);
  - *Proteus* (n = 41);
  - *Citrobacter* (n = 13);
  - *Serratia* (n = 11);
  - *Providencia* (n = 3).
- Non-fermenting bacilli: *P. aeruginosa* (n = 153);
- Rods of the genus *Acinetobacter* (n = 87);
- Gram-positive cocci (n = 211):
  - *S. aureus* (n = 104);
  - Coagulase-negative *Staphylococcus* spp. (n = 57);
  - *Enterococcus* spp. (n = 50).

The listed microorganisms were isolated from patients with invasive infections, from the following clinical specimens: urine (n = 265), pus and wound swabs (n = 257), blood (n = 242), material from the lower respiratory tract (n = 130), material from intra-abdominal infections (n = 54), cerebrospinal fluid (n = 5), and other clinical materials (n = 44). Other samples were from the upper respiratory tract (e.g., throat, pus from the ear, maxillary sinuses), wounds of the tongue, corneal and conjunctival swabs, swabs from the vestibule, cervix and amniotic fluid, ovarian cysts, and synovial fluid.

### 2.2. Microbiological Assays 

Among the tested strains, susceptible, resistant, and MDR strains were isolated. Various resistance mechanisms were identified, including extended-spectrum beta-lactamases (ESBL), carbapenemases (NDM, VIM, KPC, OXA-48), methicillin-resistant *Staphylococcus* (MRS), and high-level gentamicin-resistant (HLGR) and vancomycin-resistant *Enterococcus* (VRE). 

The agar dilution method for MIC value determination was applied as a reference method for the evaluation of fosfomycin susceptibility. As regards fosfomycin investigation, only this method is recognized by the US CLSI (Clinical and Laboratory Standards Institute), and it is also the method most recommended by EUCAST [4,12,16]. 

### 2.3. Identification of Strains and Determination of Susceptibility to Antibiotics Other Than Fosfomycin

Identification of strains and determination of their susceptibility or resistance to antibiotics other than fosfomycin were performed using an automated system—Phoenix 100^TM^ (Becton Dickinson, Sparks, MD, USA)—based on the broth microdilution method. The following panels for the Phoenix system were used for individual strains: for Gram-negative rods, NMIC-502 and NMIC/ID-402; for *Staphylococcus* spp., PMIC/ID-90. These panels also contain other antibiotics suitable for the particular microorganism. NMIC-502 and MNMIC/ID402 for bacilli, apart from fosfomycin, contain amikacin, gentamycin, imipenem, meropenem, ceftazidime, cefotaxime, cefepime, piperacillin/tazobactam, colistin, trimethoprim–sulfamethoxazole, ciprofloxacin, levofloxacin, and tigecycline. The PMIC/ID-9 panel includes antibiotics for Gram-positive strains: methicillin, gentamycin, ceftaroline, ciprofloxacin, trimethoprim-sulfamethoxazole, vancomycin, and linezolid.

### 2.4. Identification of β-Lactamases 

Various β-lactamases in rods were screened using combination disk diffusion methods (Becton Dickinson, Sparks, MD, USA). ESBL enzymes were determined by DDTs (double-disk tests) using disks with cefotaxime (30 µg), ceftazidime (30 µg), cefepime (30 µg), and amoxicillin with clavulanic acid (30 µg) (Becton Dickinson, sparks, MD, USA). MBL was determined by a double-disk synergy test (DDST) using disks with imipenem (10 µg), ceftazidime (30 µg) (Becton Dickinson, sparks, MD, USA), and EDTA (standard). KPC enzymes were identified by a combined-disk test using disks of meropenem (10 µg) and meropenem (10 µg) with 10 µL of boronic acid (GRASO BIOTECH, Starogard Gdanski, Poland) [12,17]. The identification of the various types of carbapenemases was confirmed by the commercial test RESIN-4 O.K.N.V (Coris Bioconcept, Gembloux, Belgium). Immunochromatographic lateral flow assays were used for the rapid detection of OXA-48, KPC, NDM, and VIM carbapenemases from the cultured isolates (Argenta, Poznan, Poland) [18].

A quality control of all methods was performed using the following reference strains: *Escherichia coli* ATCC 25922, *Pseudomonas aeruginosa* ATCC 27853, *Staphylococcus aureus* ATCC 29213, *Klebsiella pneumoniae* ATCC 700603 (ESBL+), and *Enterococcus faecalis* ATCC 29212 and ATCC 51299 (VRE, HLGR).

All identifications were performed in triplicate [14].

### 2.5. Diffusion Methods

A disk diffusion method on MHA plates was used to measure resistance to high concentrations of aminoglycosides in *Enterococcus* spp., using 30 µg gentamicin and 300 µg streptomycin disks (Becton Dickinson, Spark, MD, USA).

The MIC values of vancomycin, linezolid, and tigecycline for *Enterococcus* spp. were determined using the gradient diffusion method (E-test). The E-test Vancomycin VA 0.016–256 mg/L (bioMérieux Poland, Warsaw, Poland) was used to determine VRE. The following antibiotics were used to determine susceptibility to other antibiotics: E-test linezolid LZ 0.016–256 mg/L (bioMérieux Poland, Warsaw, Poland) and E-test tigecycline TGC 0.016–256 mg/L. The plates were incubated at 35 ± 2 °C for 16–20 h. Susceptibility to antibiotics was interpreted according to the EUCAST criteria [12].

### 2.6. Agar Dilution Method (Reference Method)

The agar dilution method was performed according to EUCAST Definitive Document E. Def 3.1 2000 [19]. Mueller–Hinton agar (bioMérieux Poland) was used with an addition of 25 mg/L of glucose-6-phosphate and fosfomycin (Sigma-Aldrich, Saint Louis, MO, USA). Media with fosfomycin MIC ranges from 0.064 mg/L to 512 mg/L and a final bacterial inoculum of 1 × 10^4^ CFU/spot were used [19]. Due to spreading growth on the agar surface, in the case of *Proteus* spp. strains, 24-well microtiter plates with successive agar concentrations of fosfomycin were used for individual strains, similar to the certified commercial method [11]. The plates were incubated at 35 ± 2 °C for 16–20 h. The lowest concentration of the antibiotic in the agar that completely inhibited bacterial growth was regarded as the MIC value for fosfomycin. In Figures S1 and S2 of the Supplementary Materials, the method of reading MIC values is presented. In the interpretation of the results, the EUCAST criteria [12] were implemented with MIC breakpoints (mg/L), as follows:

For *Enterobacterales*: S ≤ 32; R > 32;

For *Staphylococcus* spp.: S ≤ 32; R > 32;

For *Pseudomonas aeruginosa*: S ≤ 128; R > 128.

For strains of *Acinetobacter* spp. and *Enterococcus* spp. for which the criteria for clinical interpretation are not currently established, only MIC values are provided in this study.

### 2.7. Statistical Analysis

Statistical tests were performed using the SciPy Library in the Python programming language. The differences in susceptibility between the groups (i.e., strains, materials, and resistance mechanisms) were compared using the chi-squared test for independent variables. The same methodology was implemented for all of the groups compared. The test results were considered significant for *p*-values < 0.05. More statistical details are provided in Table S1 and in Section S1 of the Supplementary Materials.

## 3. Results

The susceptibility of 997 bacterial strains to IVFOS was determined, including the genus *Acinetobacter* and *Enterococcus* spp., for which there are no interpretation criteria (i.e., BPs) in the current EUCAST recommendations [12]. Therefore, for these two, only the analysis of MIC values was performed. In Table 1, the resistance mechanisms of the tested strains are presented. In the group of *Enterobacterales*, the dominant resistance mechanism was ESBL production (175 strains, including 26 *Klebsiella* strains that were additionally characterized by production of carbapenemases). The *K. pneumoniae* strains dominated in the group of carbapenemase producers, of which 71 were found to be metallo-β-lactamases (NDM), 4 were metallo-β-lactamases (VIM), and 7 were serine-β-lactamases (OXA-48). In addition, carbapenemases were found in 12 strains of *P. aeruginosa* (mainly VIM) and in individual strains of *E. coli* and *Enterobacter* spp. Among multidrug-resistant strains of *Acinetobacter* spp., only one strain produced metallo-β-lactamases, whereas in the case of other strains no mechanisms were identified with the use of phenotypic methods.

Among all of the tested strains, 83% were susceptible to IVFOS, excluding *Acinetobacter* and *Enterococcus* species (Table 2). The largest percentage of susceptible strains was found among *S. aureus* and *E. coli*. The smallest percentage of sensitive strains (66%) was found in the *Klebsiella* spp. group. It should be noted that in *Klebsiella* spp. and among *E. coli* strains, MDR strains were identified (68.8% and 19.3%, respectively). In the group of *S. aureus*, 38.5% were MRSA strains, which is relevant for data analysis (Table 1). Significant statistical differences in strain susceptibility (*p*-value < 0.05) were found for most of the analyzed pairs, including the sensitivity of *Klebsiella* spp. (KL) vs. *E. coli* (EC), KL vs. *Proteus* spp. (P), KL vs. other, KL vs. *Pseudomonas* spp. (PS), KL. vs. *S. aureus* spp. (SA), *E. coli* vs. *Enterobacter* spp. (E), *E. coli* vs. CNS, E vs. other, E vs. PS., E vs. SA, P. vs. SA, other vs. CNS, PS. vs. SA, PS. vs. CNS, and SA vs. CNS.

The susceptibility analysis of strains according to the site of isolation indicated significant statistical differences (*p*-value < 0.05) between urine and pus and wound swabs, and between blood isolates and pus and wound swabs (Table 3). Strains isolated from blood were the least sensitive (77.8%). Only three strains were isolated from cerebrospinal fluid (one *E. cloacae* ESBL+, one MRCNS, and one MSCNS), but because of the clinical significance of this material it was included in the investigation. All of these three strains were susceptible to IVFOS.

IVFOS is recommended primarily for the treatment of infections caused by MDR strains. Therefore, susceptibility to this antibiotic was also determined in the group of ESBL+, carbapenemase-positive strains producing at least one of MBL enzymes (e.g., NDM-1, VIM), KPC, or OXA-48, as well as in the group of MDR Gram-negative strains that did not produce the abovementioned β-lactamases and in the group of MRS strains. The results are shown in Table 4. Statistically significant differences (*p*-value < 0.05) were shown for the following pairs: susceptible vs. ESBL, susceptible vs. ESBL + MBL/KPC/OXA-48, susceptible vs. MBL/KPC/OXA-48, ESBL vs. MBL/KPC/OXA-48, MBL/KPC/OXA-48 vs. other MDR, MBL/KPC/OXA-48 vs. MRS, and ESBL + MBL/KPC/OXA-48 vs. MRS.

According to the obtained results, strains that were sensitive to other antibiotics were also fosfomycin-susceptible. IVFOS showed the lowest sensitivity in the group of Gram-negative carbapenemase-positive strains and those that additionally produced ESBL enzymes—55.6% and 61.6%, respectively. Among ESBL+ strains, 75% susceptibility to IVFOS was found (Table 4).

MIC evaluation indicated that the lowest MIC_50_ and MIC_90_ values were found for *S. aureus* and *E. coli* (0.5 mg/L and 1 mg/L; 4 mg/L and 32 mg/L, respectively) (Table 5). Conversely, the highest MIC_50_ values were found for *Acinetobacter* spp. (256 mg/L), while the highest MIC_90_ values were found for *Acinetobacter* spp. and *Klebsiella* spp. (256 mg/L and 512 mg/L, respectively).

**Figure 1:**
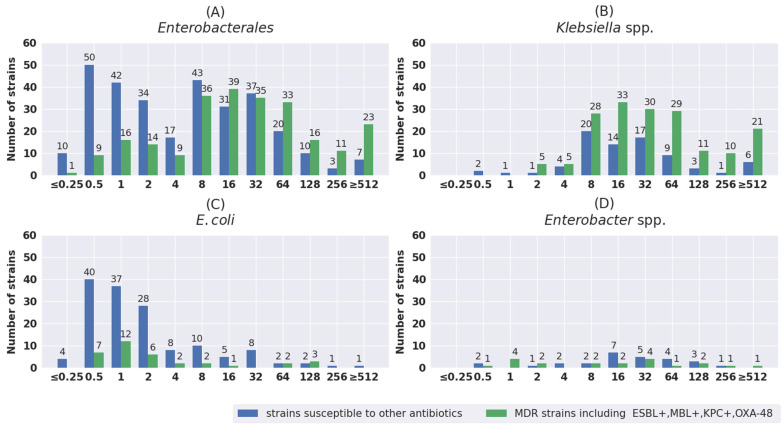
Distribution of MIC values of fosfomycin for total *Enterobacterales* (**A**), *Klebsiella* spp. (**B**), *E. coli* (**C**), and *Enterobacter* spp. (**D**).

**Figure 2:**
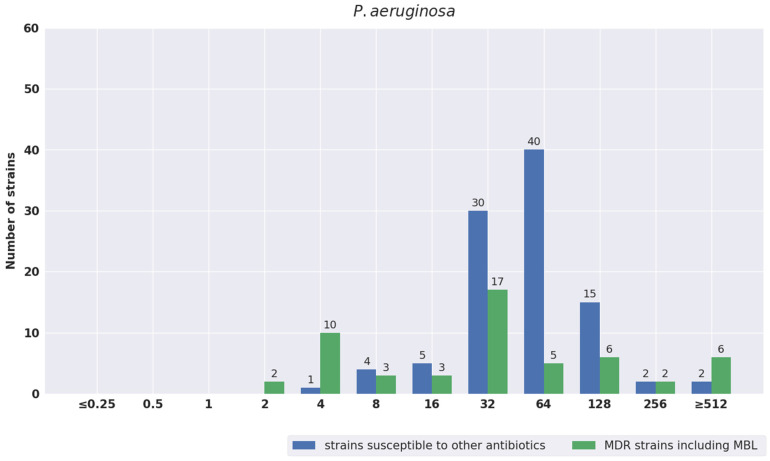
Distribution of MIC values of fosfomycin for *P. aeruginosa*.

**Figure 3:**
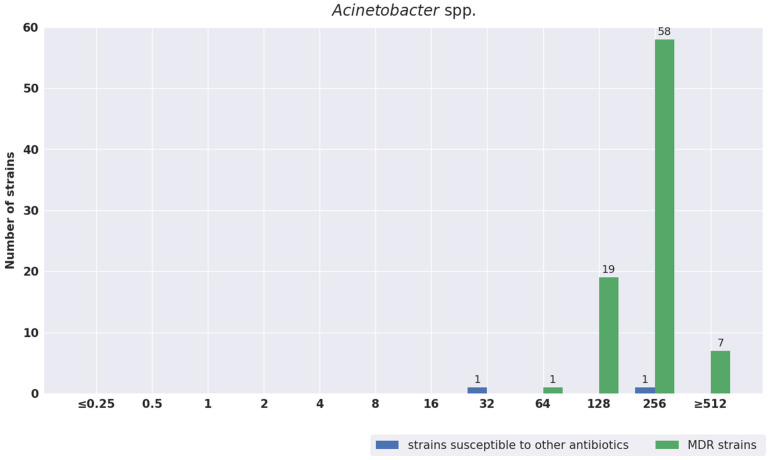
Distribution of MIC values of fosfomycin for *Acinetobacter* spp.

**Figure 4:**
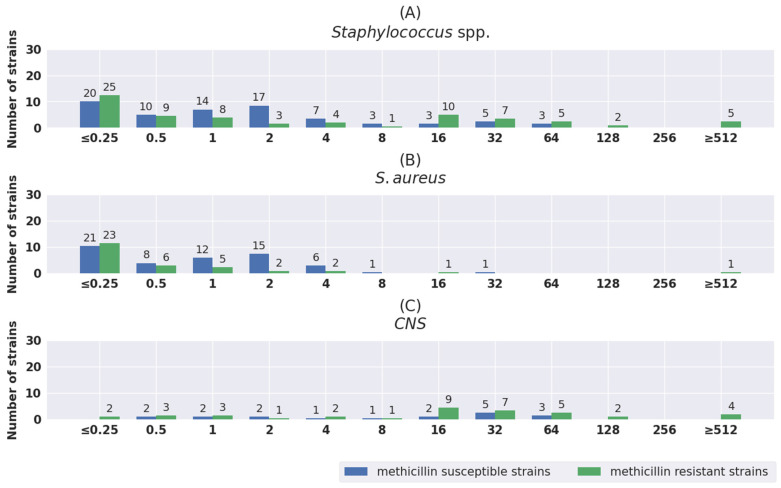
Distribution of MIC values of fosfomycin for total *Staphylococcus* spp. (**A**), *S. aureus* (**B**), and coagulase-negative *Staphylococcus* (CNS) (**C**).

**Figure 5:**
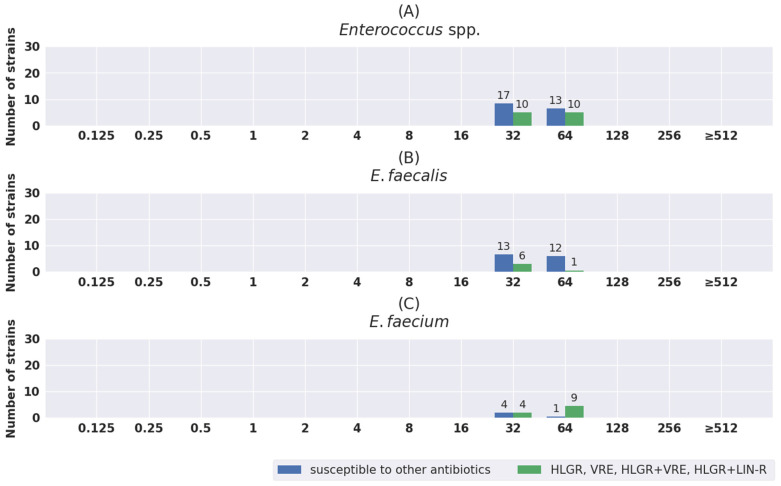
Distribution of MIC values of fosfomycin for total *Enterococcus* spp. (**A**), and separately for *E. faecalis* (**B**) and *E. faecium* (**C**).

The distribution of MIC values was also analyzed separately for susceptible strains and for strains with different antibiotic resistance mechanisms. The results are shown in Figure 1, Figure 2, Figure 3, Figure 4 and Figure 5. The tested strains displayed a wide range of MIC values—especially *Enterobacterales* rods—regardless of the presence or absence of resistance mechanisms (≤0.25 mg/L–≥512 mg/L).

*Acinetobacter* spp strains displayed a narrow range of MIC values, from 32 mg/L for one sensitive strain to more than 512 mg/L for resistant strains. For *Enterococcus* spp., the MIC values ranged from 32 mg/L to 64 mg/L.

The lowest MIC values were exhibited by *S. aureus* strains—both MSSA and MRSA. IVFOS MIC values of no more than 0.25 mg/L (21/64 MSSA and 23/40 MRSA) predominated in both groups. 

Analysis of the MIC values for *Enterobacterales* provided no clear conclusions. However, in the largest group of the tested strains—namely, *Klebsiella* spp.—it was noted that the majority of both sensitive and resistant rods showed MIC values in the range from 8 mg/L to 64 mg/L, accounting for 76.9% (60/78) and 69.8% (120/172), respectively. In the range from 8 mg/L to 32 mg/L (the BP for susceptible strains according to EUCAST v.12), the respective values were 65.4% and 52.9%.

However, more strains with MICs > 32 mg/L and ≥ 512 mg/L were observed among resistant versus sensitive strains—41.3% vs. 24.1% and 12.2 vs. 7.7%, respectively. It should be noted that strains with the highest MIC values of at least 512 mg/L were found not only in groups of drug-resistant bacteria, but also in sensitive bacteria: *Enterobacterales* (seven strains) and *P. aeruginosa* (two strains). Compared to other species tested, in the case of *P. aeruginosa*, more strains with a low IVFOS MIC ≤ 32 mg/L were observed in the resistant group than in the sensitive group—64.8% (35 strains/54) vs. 40.4% (40/99).

Table 6 and Table 7 present the susceptibility of the tested strains to antibiotics other than IVFOS. Among *Enterobacterales* and *P. aeruginosa* strains suceptible to IVFOS, 100% susceptibility to other antibiotics was not found. Simultaneously, full sensitivity to no other antibiotics was demonstrated. Most of the abovementioned strains were susceptible to both IVFOS and amikacin (91.4% and 83.5%, respectively), to imipenem and meropenem (more than 85% among *Enterobacterales*), and to meropenem and piperacillin with tazobactam (around 75% among *P. aeruginosa*). Only 6% of *Acinetobacter* spp. strains were sensitive to all antibiotics.

In the group of *S. aureus,* 100% fosfomycin-susceptible strains were observed, and 38% were MRSA strains. All strains tested were susceptible to vancomycin and linezolid. One fosfomycin-resistant strain was also resistant to methicillin, gentamicin, ciprofloxacin, and trimethoprim–sulfamethoxazole.

Among fosfomycin-susceptible CNS, about 65% were MRCNS and, at the same time, more than 90% were susceptible to vancomycin and linezolid. Fosfomycin-resistant strains also remained susceptible to vancomycin (100%) and to linezolid (about 85%). All of the studied *Enterococcus* spp. strains were susceptible to tigecycline, and 98% were susceptible to linezolid.

## 4. Discussion

In the current decade, the treatment of bacterial infections poses a serious clinical problem. In order to achieve therapeutic success, a number of conditions related to the drug, pathogen, and interactions in the patient’s body must be fulfilled at the same time. Changes in patients’ pharmacokinetic parameters impede the penetration of antibiotics to the site of infection and the achievement of therapeutic concentrations. Good penetration into various organs and systems is a crucial feature required of antibiotics. Therefore, antibiotics such as β-lactams—including penicillin, cephalosporins (especially high-generation ones), carbapenems, and fluoroquinolones—have become some of the most commonly used in treatment. However, good drug pharmacokinetics is not sufficient for effective therapy. Moreover, susceptibility of pathogens to the selected antibiotic is essential. Here, bacteria have been setting the bar higher and higher. Selection of strains resistant not only to a group, but to several groups or even to all available antibiotics, is taking place more frequently and rapidly. According to the WHO and ECDC’s 2020 data, worldwide—and in Europe in particular—Gram-negative rods are the most resistant to antibiotics. In 21 European countries, including Russia, Belarus, Ukraine, and Turkey, more than 50% of *Acinetobacter* spp. strains are resistant to carbapenems [20]. The ECDC’s detailed data indicate that in countries such as Croatia, Romania, and Lithuania this percentage exceeds 90%, while in Italy, Poland, and Bulgaria it is about 80% [21]. Unfortunately, *Acinetobacter* species have been reported to show resistance to colistin, which is regarded as the antibiotic of last resort [22,23,24]. Research from Saudi Arabia has shown close to 9% prevalence of such strains, and research from Greece has shown 42% prevalence of colistin-resistant strains in the group of 31 *Acinetobacter* spp. strains responsible for sepsis [22,23]. Serious concerns are caused by reports on the identification of strains that are resistant to the newest antibiotic—cefiderocol, which has been regarded as an option in cases where the pathogen is resistant to all other antibiotics [25,26,27].

Resistance to at least three groups of antibiotics observed in more than 50% of *Acinetobacter* spp. was recorded in 8 EU countries, reaching the highest values in Greece (90.8%). *K. pneumoniae* strains—more than 50% of which were resistant to the third generation of cephalosporins in 18 European countries, and similar percentages of which were resistant to carbapenems in 6 countries (Belarus, Georgia, Greece, Moldova, Russia, and Ukraine)—are also a huge problem [20]. European data provided by the ECDC indicated that simultaneous resistance of *Klebsiella* spp. to three groups of antibiotics—i.e., third-generation cephalosporins, fluoroquinolones, and aminoglycosides—was demonstrated in Greece and Bulgaria (more than 50%), as well as in Slovakia, Poland and Romania (around 44–48%).

Resistance to antibiotics has also been on the rise among *P. aeruginosa* strains. In 2020, more than 50% of these rods were resistant to carbapenems in 6 countries, and over 25% resistance was found in 14 countries. Resistance of more than 25% of *P. aeruginosa* strains to ceftazidime, or to at least three groups of antibiotics among such drugs as piperacillin with tazobactam, fluoroquinolones, ceftazidime, aminoglycosides, and carbapenems, was found in four EU countries. Regardless of the genus or species of Gram-negative rods, the phenomenon of rapidly increasing resistance to fluoroquinolones is also well known. More than 50% prevalence of resistant *Acinetobacter* spp. strains was found in nine EU countries, in the case of *Klebsiella* spp. in seven countries, and for *P. aeruginosa* in one country [21].

The abovementioned data, along with the experience of physicians who review antibiogram results on a daily basis, leave no doubt that the treatment of infections with beta-lactams or fluoroquinolones is becoming less feasible. Other antibiotics are therefore needed to replace the most commonly used ones.

Cephalosporins, carbapenems, and fluoroquinolones possess a broad spectrum of antimicrobial activity and good penetration into organs—a vital feature in empirical therapy. Therefore, the loss of these antibiotics due to increasing bacterial resistance is also a huge loss for modern medicine. It is not easy to find comparable substitutes for these antibiotics. In the age of multidrug resistance, colistin is being used again, despite poor organ penetration and a narrow spectrum of antimicrobial activity. Tigecycline has a broad spectrum of antimicrobial activity (excluding *P. aeruginosa*) and achieves effective concentrations in tissues, but unfortunately not in blood. Treatment of general infections with this antibiotic is therefore not advisable.

IVFOS has a broad spectrum of antimicrobial activity and very good penetration into many systems and tissues. It is also recommended for the treatment of many severe infections in both children and adults, as confirmed by many publications. In a prospective multicenter study of 209 adult patients from 20 ICU units in Germany and Austria, Putensen et al. evaluated the clinical efficacy of intravenous fosfomycin. Its use in combination therapy in various infections—including lung, CNS, urinary tract, skin and soft tissue, intra-abdominal, and other infections—brought clinical success in 81.3% of cases and a success rate of 84.8% in the case of infections caused by MDR strains [28]. In another cohort study from India, the authors obtained an overall clinical efficacy of 55% when IVFOS was used in ICU patients, but significantly better results (i.e., success rate of 79.24%) were noted in the case of UTI treatment [29]. Pontikis et al. observed the efficacy of fosfomycin in 54.2% of ICU patients, mainly with bloodstream infections (BSIs) and ventilator-associated pneumonia (VAP) [30]. In turn, Tsegka et al. analyzed the available reports on the efficacy of IVFOS in the treatment of central nervous system infections mainly caused by *Staphylococcus* spp. strains, *Streptococcus pneumoniae*, *Neisseria meningitidis*, and others [31]. The authors evaluated the antibiotic’s efficacy at nearly 94%. In another article, Tsegka et al. also highlighted the significance of IVFOS in therapy for bone and joint infections [32].

In infections of orthopedic implants and devices, the formation of bacterial biofilm and survival of bacteria within osteoblasts are of crucial importance [33]. Stracquadanio S. et al. pointed to the fact that *S. aureus* has a special ability of internalization in non-professional phagocytes, while in *S. epidermidis* or *S. lugdunensis* this ability is much lower [33]. Through internalization in osteoblasts, bacteria become inaccessible to immune system cells and to most antibiotics [33]. In such cases, fosfomycin’s particle size and its ability to penetrate into bacterial biofilms and osteoblasts have a significant impact on its antimicrobial effectiveness [34,35,36]. An analysis of data from 19 papers conducted by Tsegka et al. showed 82.2% efficacy of IV fosfomycin in the treatment of bone and joint infections, most of which were caused by *S. aureus* (38.9%) [32]. 

Although IVFOS is authorized for the treatment of children and neonates, there are only a few reports about its efficacy in these age groups. Li at al. analyzed five studies on the use of IVFOS in neonatal sepsis. The effectiveness of treatment in combination therapy was between 88% and 100% [37].

Another author, Williams, also looked at the use of fosfomycin in children and, in addition to the five studies mentioned above, he also included two additional studies conducted by Baquero et al. and Corti et al. [10,38,39]. In the first study, from 1977, the authors used fosfomycin in the treatment of infections caused by *Serratia marcescens*. Clinical efficacy was achieved in only 50% of cases when the antibiotic was used in monotherapy, and in 90% of cases when it was used in combination with gentamicin or carbenicillin [38]. In the second study, 23 children with acute hematogenic osteomyelitis were given IVFOS as monotherapy, and full clinical success was achieved [39]. However, it should be stressed that due to rapid selection of resistant subpopulations of strains, IVFOS is not recommended for monotherapy.

As indicated above, fosfomycin is suitable for the treatment of many infections caused by Gram-positive and Gram-negative bacteria, both in children and adults.

In this study, we analyzed the in vitro IVFOS susceptibility of clinical strains isolated from various types of infections (the age of the patients was not taken into consideration). Except for *Acinetobacter* spp. and *Enterococcus* spp., the tested strains were isolated from urine (240), pus and wound swabs (238), and blood (203). Susceptibility to IVFOS was shown in 89% of strains isolated from skin and soft tissue infections, while the figure for strains isolated from lower respiratory tract and intra-abdominal infections was approximately 85%. The lowest level of susceptibility (78–79%) was displayed by strains isolated from urine and blood, with no statistical significant differences between these groups. In 2009–2010, scientists from Beijing assessed the sensitivity of *E. coli* isolates and concluded, based on then-current interpretation criteria (EUCAST v.12), that strains isolated from urine were the most sensitive to fosfomycin (94.7%), followed by strains isolated from blood (92.4%) and from purulent lesions (91.3%), with strains isolated from sputum being the least susceptible (85.6%) [40]. In our study, these rates were slightly lower, probably because the results concerned total strains of different types, including the much more resistant *Klebsiella* spp. and *P. aeruginosa* strains. In addition, in the study of Li et al., the strains tested were ESBL+, while the strains in our investigation also showed the presence of carbapenemases [40]. Another study from India, performed on 2229 strains, confirmed 95% fosfomycin susceptibility in strains isolated from urine, although MDR and carbapenemase-positive bacteria were already included in this study [41].

In our study, irrespective of the strains’ origin, 83% were susceptible to IVFOS, of which 43.3% possessed at least one resistance mechanism. The highest sensitivity (over 90%) was determined for *S. aureus, E. coli*, and *P. aeruginosa*, while *Klebsiella* spp. were the least susceptible strains (66%). A high proportion of susceptibility to IVFOS in *E. coli* and *S. aureus* populations is widely reported in the global literature. The aforementioned study by Li et al. found 91.1% sensitivity among *E. coli* strains isolated from various infections [40]. Czech and Italian scientists described IVFOS susceptibility among strains isolated from urine (97% and 96.9%, respectively) [42,43]. Similarly, in the case of *S. aureus*, more than 90% susceptibility to IVFOS is usually observed [44,45,46].

Additional properties of fosfomycin—such as activity against intracellular pathogens, antimicrobial potential against Panton–Valentine leucocidin (PVL) produced by staphylococci, and the possibility of immunomodulation of the antimicrobial activity of neutrophils—are also desirable and can enhance its activity in the treatment of severe infections [35,47,48,49,50,51]. Although fosfomycin is not the first choice for treating infections caused by MSSA or MRSA strains, these additional properties should be taken into account, especially considering that almost 100% sensitivity to this antibiotic is observed (Table 7).

Valour et al. conducted an evaluation of the intra-osteoblastic activity of various antibiotics. Survival of *S. aureus* in osteoblasts is known to be one of the factors leading to the recurrence of bone and joint infections [33,35]. The authors demonstrated that fosfomycin shows an intracellular bactericidal effect similar to those of linezolid, ofloxacin, rifampicin, oxacillin, and clindamycin. Vancomycin and daptomycin do not display such properties, while ceftaroline and teicoplanin have only bacteriostatic effects [35]. Fosfomycin may also limit the amount of small-colony variants (SCVs) of *S. aureus*, although maximal antibiotic doses are required for this effect, and far better results have been achieved using ofloxacin [35]. Zelmer et al., on the other hand, pointed to the low activity of fosfomycin against extracellular small-colony variants (SCVs) of *S. aureus* and, consequently, to its limited usefulness in the treatment of chronic bone infections [34].

Susceptibility to fosfomycin is slightly different in the case of *P. aeruginosa*. Firstly, unlike most bacteria, the disodium salt of fosfomycin enters the cell by means of active transport using only one transporter—glycerol-3-phosphate—due to the lack of glucose-6-phosphate (UhpT), as demonstrated by Castaneda-Garcia et al. [52]. Secondly, no BPs have been established to allow clinical interpretation of the MIC values obtained for fosfomycin and *P. aeruginosa.* At the same time, EUCAST has provided the BP for wild-type (WT) *P. aeruginosa*. A WT is a strain that does not have an acquired antibiotic resistance mechanism detected by phenotypic methods. It has been characterized by an MIC that does not exceed the defined cutoff point known as the ECOFF (epidemiological cutoff value) for the pathogen and antibiotic [53]. Therefore, the ECOFF value for fosfomycin (MIC ≤ 128 mg/L) presented on the EUCAST website has become the basis for many authors’ assessment of the susceptibility or resistance of *P. aeruginosa* strains [12]. However, in view of the consultations announced by EUCAST, it is not certain whether the ECOFF value for *P. aeruginosa* will be maintained [15].

The withdrawal of the ECOFF value from EUCAST guidelines would mean that fosfomycin would not be included in antibiograms. This might be unfavorable in the case of infections caused by *P. aeruginosa*, as fosfomycin—in combination with other antibiotics—is one of the important therapeutic options for MDR strains [54,55,56,57,58]. In combination therapy, antibiotics are selected mainly based on the sensitivity of the strains causing infections. Therefore, susceptibility assessment is important for such a treatment. Our research found that among *Pseudomonas* spp., almost 91% of strains displayed sensitivity. Considering the ECOFF, Walsh et al. reported 76.6% sensitivity among *P. aeruginosa* strains isolated from 64 patients—mainly from cystic fibrosis [59]. Zhanel et al. did not precisely specify the sensitivity percentage but gave the MIC_90_ for fosfomycin as 128 mg/L [4]. It was concluded that at least 90% of *P. aeruginosa* strains were susceptible to IVFOS. In studies published in 2021, 86.6% of tested strains were sensitive, while 96% susceptibility of strains isolated from children with sepsis was reported by Williams et al. [10,60]. In a multicenter study performed in 2013, researchers reported 86.4% susceptibility among the tested strains, while the results of an Indian study performed in 2019 reported only 50% susceptibility of the tested stains [61,62].

*Klebsiella* spp. Strains—especially *K. pneumoniae*—are currently considered to be the most resistant bacteria in the world [63,64]. Therefore, fosfomycin is mentioned as a potential therapeutic option against pathogens that are resistant to many other antibiotics, including beta-lactams. In its new 2022 recommendations, the European Society of Clinical Microbiology and Infectious Diseases (ESCMID) included the possibility of implementing IVFOS in combination therapy for patients with severe infections caused by carbapenem-resistant *Enterobacterales* that are susceptible to polymyxins, aminoglycosides, tigecycline, or fosfomycin, or in the case of unavailability of antibiotics combined with β-lactam inhibitors [55]. 

Furthermore, in the report of the British Society for Antimicrobial Chemotherapy, fosfomycin was noted as the last-resort drug for MDR infections [65]. The authors of these recommendations further specified IVFOS as an alternative to carbapenems for use against ESBL+ strains. In our research, only 66% of *Klebsiella* spp. strains were susceptible to fosfomycin. However, it is relevant that in this group, 170 out of the 250 tested strains produced β-lactamases, of which 84 were carbapenemases. All NDM-1-positive strains were of the *Klebsiella* genus. Resistant strains were predominant in this type, which may explain their lower susceptibility to fosfomycin compared to the other bacterial types tested. An analysis of MIC value distribution indicated that among resistant *Klebsiella* spp. strains, 41.3% had MIC values > 32 mg/L, whereas among sensitive strains such MIC values were observed at a rate of 24.4%.

The analysis of susceptibility to IVFOS according to the type of resistance mechanism to other antibiotics indicated that strains producing only carbapenemases or ESBL enzymes (55.6–61.6%) were the least sensitive. ESBL strains were almost 76% sensitive to IVFOS, while MDR strains with unidentified resistance mechanisms were sensitive in about 82% of cases. These results indicate a high probability of infections caused by fosfomycin-sensitive *Enterobacterales* and potential application of fosfomycin in not only targeted but also empirical therapy. However, each empirical antibiotic administration should be verified by a microbiological test result.

Studies from Poland conducted in 2011–2020 showed practically the same susceptibility levels as those demonstrated in the present paper, both in the carbapenemase-positive (55%) and carbapenemase-negative strains (75%), as well as among *Klebsiella* spp. (65%) [66]. When comparing these outcomes, it could be concluded that resistance to fosfomycin does not increase. However, such thinking can be misleading, as it should be taken into account that in Poland IVFOS has been approved since Q3 of 2019. The analysis of the relationship between its use and the pace of resistant strain selection will be possible in subsequent years. 

Turkish researchers studied *E. coli* and *K. pneumoniae* strains producing OXA-48, NDM-1, VIM, and IMP enzymes and identified 56.3% susceptibility to fosfomycin [67]. This result is consistent with the outcomes presented in this study. Increased sensitivity rates in the group of carbapenemase-positive strains of *Enterobacterales* were reported for KPC and NDM strains (84.2% and 92.9%, respectively) [41]. Based on current criteria for the interpretation of MIC values (sensitive strains ≤ 32 mg/L), the SENTRY global surveillance program also reported 82.6% susceptibility among 23 *E. coli* and *K. pneumoniae* carbapenemase-positive strains tested [68].

Undoubtedly, monotherapy with IVFOS accelerates the development of resistance; therefore, combination therapy is recommended [55]. Unfortunately, no advantage has been shown for the use of IVFOS in combination therapy. However, there are literature reports on the use of IVFOS in various combinations with antibiotics such as colistin, β-lactams, ceftazidime–avibactam, aminoglycoside, fluoroquinolones, tigecycline, and even rifampicin, depending on the pathogens and their susceptibility profiles [55,69,70,71,72,73,74].

The level of susceptibility to any antibiotic, including to fosfomycin, is not constant over time and must be monitored on an ongoing basis, even during daily microbiological diagnostics. The recommendation of the agar dilution method certainly does not support the testing procedures, as it is difficult and time-consuming. 

The distribution of the presented MIC values shows that both multidrug-resistant strains and strains that are susceptible to other antibiotics can display a wide range of MIC values, as can be seen in the case of *Enterobacterales* or *P. aeruginosa*. MDR *Enterobacterales* strains may show very low MIC values, such as 0.25 or 0.5 mg/L, but strains that are sensitive to other antibiotics may present a high level of resistance to fosfomycin, with MIC values as high as ≥512 mg/L. However, it was demonstrated that in the group of strains sensitive to other antibiotics, susceptibility to IVFOS was greater (90.8%) than in the group of MDR strains (72.9%). A similar observation was made with regard to the quantity of strains with low MIC values. MDR strains may have very low MIC values for fosfomycin, and in such cases the risk of rapid selection of resistant strains is delayed in time, making effective treatment more probable. 

A separate discussion is required to describe the role of fosfomycin in infections caused by *Enterococcus* spp. and *Acinetobacter* spp. It is not possible to assess the sensitivity to IVFOS due to the lack of interpretation criteria. In our study, for *Acinetobacter* spp., we obtained high MIC values indicating resistance to fosfomycin, but such values are commonly identified [75,76]. Currently, infections caused by *Acinetobacter* are probably one of the biggest therapeutic challenges. New antibiotics, such as ceftazidime–avibactam, meropenem–vaborbactam, or plazomicin, are not active against *Acinetobacter* spp. [77]. There is also no certainty that cefiderocol—the most recent antibiotic—will be effective against MDR *Acinetobacter* spp., given the disturbing reports about strains that are resistant to this antibiotic [25,26,27]. The main mechanism behind the resistance of *Acinetobacter spp.* to cefiderocol is reduction in the expression of the siderophore receptor genes *pirA* and *piuA* [25,26,27]. Therefore, studies on fosfomycin’s activity in combination with other antibiotics are being conducted, regardless of the high MIC values reported for *Acinetobacter* spp. Nwabor et al. evaluated the effectiveness of fosfomycin combinations with selected antibiotics, such as carbapenems and aminoglycosides. The MIC_90_ of IVFOS for the tested strains was 256 mg/L, and the strains were also carbapenem-resistant [78]. This is also consistent with our results. A synergistic effect with gentamicin was identified, and an additive effect was observed for other antibiotics. A 2–16-fold reduction in the MIC values of the tested antibiotics was noted when they were combined with fosfomycin [76]. Another study proved fosfomycin’s synergism with imipenem against *Acinetobacter* spp. [75]. Despite the above reports, in the most recent recommendations of the ESCMID and ESICM (the European Society of Intensive Care Medicine) in December 2021, fosfomycin was not included as a therapeutic option against *Acinetobacter* spp.—even in combination with other antibiotics [55]. However, it was present as part of combination therapy in treatment regimens proposed by Bassetti and Garau in 2021 [79].

The possibility of using fosfomycin in enterococcal infections has been triggering even more discussion. EUCAST does not recommend an assessment of enterococcal susceptibility to either oral or intravenous fosfomycin. Studies have shown its potential activity with regard to *E. faecalis* in particular, but also *E. faecium* [80,81,82]. These strains usually present MIC values in a rather narrow range of concentrations, as confirmed by the results obtained in our research. The MIC values were in the range from 32 mg/L to 64 mg/L, both for VRE/HLGR and for susceptible strains; the MIC_50_ and MIC_90_ for *E. faecalis* and *E. faecium* were 32 mg/L and 64 mg/L and 64 mg/L and 64 mg/L, respectively. Similar MIC values were obtained by other authors, although for *E. faecium* they were slightly higher, reaching 128 mg/L [4,68,80]. There have also been studies where the MIC reached values of >1024 mg/L. This was the case for the VRE strains tested by Guo et al. [81]. In a group of 234 VRE strains, the authors reported only 4.3% with an MIC of 32 μg/mL, 39.3% with 64 μg/mL, and 56.4% with ≥128 mg/L [81]. Usually, according to the analysis of the literature data by Vardakas et al., MIC values for *Enterococcus* spp. do not exceed 128 mg/L [82]. However, further research is needed on the applicability of fosfomycin in the treatment of infections caused by *E. faecium*, where in the case of GRE, HLAR, and linezolid-resistant strains, the therapeutic range is dramatically limited. Even new antibiotics such as eravacycline do not have indications as broad as those of fosfomycin.

## 5. Conclusions

A high percentage of susceptibility to fosfomycin in a group of bacteria without multidrug resistance features and more than 70% sensitivity among strains with different resistance mechanisms may indicate the usefulness of this antibiotic in the treatment of various infections.

A broad antimicrobial spectrum and good penetration into the organs allow fosfomycin to be used in the treatment of mixed infections and to be applied in empirical therapy. Currently, a lack of its activity against *Acinetobacter* spp. should be assumed, but in the case of *Enterococcus* spp. the obtained MIC values seem not to presume the absence of such activity against certain strains. Much greater caution is required when fosfomycin is prescribed when carbapenemase-positive strains are the suspected cause of infection. The sensitivity of these strains to fosfomycin at a rate slightly above 50% makes it necessary to confirm their susceptibility in microbiological tests before administration. It is worth stressing that among *Enterobacterales* and *P. aeruginosa* that were fully susceptible to fosfomycin, 100% susceptibility to other antibiotics was not found. The obtained distribution of the MIC values can contribute to increasing awareness of susceptibility to fosfomycin, and it may be used to establish breakpoints not only for *E. coli* and *S. aureus*—as planned by EUCAST—but also for *P. aeruginosa* and *Enterobacterales*.

## Figures and Tables

**Table 1:** Resistance mechanisms of the studied clinical strains.

Bacterial Strains	Number of Strains	S *	Only ESBL *	ESBL + MBL/KPC/OXA-48 *	Only MBL/KPC/OXA-48 *	Other MDR *	MRS *	Only VRE *	Only HLGR	VRE + HLGR	HLGR + LIN-R
Total	997	519	149	26	82	122	79	6	8	6	
*Enterobacterales*	546	306	149	26	60	5					
*Klebsiella* spp.	250	78	86	26	58	2					
*E. coli*	181	146	34		1						
*Enterobacter* spp.	47	27	17		1	2					
*Proteus* spp.	41	30	10			1					
*Citrobacter* spp.	13	11	2								
*Serratia* spp.	11	11									
*Providencia* spp.	3	3									
*Pseudomonas* spp.	153	99			21	33					
*Acinetobacter* spp.	87	2			1	84					
*Staphylococcus* spp.	161	82					79				
*S. aureus*	104	64					40				
CNS	57	18					39				
*Enterococcus* spp.	50	30						6	7	6	1
*E. faecalis*	32	25							5	1	1
*E. faecium*	18	5						6	2	5	

* S: Susceptible strains (not MDR), including ESBL, KPC, MBL, OXA-48, MRS, VRE, HLGR, and Lin R; MBL/KPC/OXA-48: at least one of the carbapenemases; ESBL: extended-spectrum beta-lactamase; MBL: metallo-β-lactamase; MRS: methicillin-resistant *Staphylococcus*; VRE–vancomycin-resistant *Enterococcus*; HLGR: high-level gentamicin-resistant *Enterococcus*; Lin-R: resistant to linezolid; CNS: coagulase-negative *Staphylococcus*.

**Table 2:** Susceptibility to IVFOS (except for *Enterococcus* spp. and *Acinetobacter* spp. strains).

Bacterial Strains	n	Susceptibility to IVFOS	
		n	%
Total	860	714	83.00%
Total *Enterobacterales*	546	429	78.60%
*Klebsiella* spp.	250	165	66.00%
*E. coli*	181	170	93.90%
*Enterobacter* spp.	47	34	72.30%
*Proteus* spp.	41	34	82.90%
other	27	26	96.30%
*Pseudomonas* spp.	153	139	90.80%
Total *Staphylococcus* spp.	161	146	90.70%
*S. aureus*	104	103	99.00%
CNS	57	43	75.40%

**Table 3:** Susceptibility to IVFOS depending on the source of the examined strains (except for *Acinetobacter* spp. and *Enterococcus* spp.).

Sources of Strains– Clinical Samples	n	Susceptibility to IVFOS	
		n	%
Urine	240	190	79.20%
Pus and wound swabs	238	211	88.70%
Blood	203	158	77.80%
From lover respiratory tract	97	83	85.40%
Intra-abdominal samples	43	37	86.00%
Other	36	32	88.90%
Cerebrospinal fluid (CSF)	3	3	100.00%

**Table 4:** Susceptibility to IVFOS among strains (except *Acinetobacter* spp. and *Enterococcus* spp.) that were susceptible and resistant to other antibiotics.

Mechanism of Resistance	n	Susceptibility to IVFOS	
		n	%
Susceptible	487	442	90.80%
Only ESBL	149	113	75.80%
Only MBL/KPC/OXA-48	81	45	55.60%
ESBL + MBL/KPC/OXA-48	26	16	61.60%
Other MDR Gram-negative rods	38	31	81.60%
MRS	79	67	84.80%

**Table 5:** MIC_50_ and MIC_90_ values of fosfomycin.

Bacterial Strains	Values (mg/L)	
	MIC_50_	MIC_90_
Total *Enterobacterales*	8	128
*Klebsiella* spp.	32	512
*E. coli*	1	32
*Enterobacter* spp.	16	128
*Proteus* spp.	8	64
Other *Enterobacterales*	2	32
*Acinetobacter* spp.	256	256
*Pseudomonas* spp.	64	128
Total *Staphylococcus* spp.	1	32
*S. aureus*	0.5	4
CNS	16	64
Total *Enterococcus* spp.	32	64
*E. faecalis*	32	64
*E. faecium*	64	64

**Table 6:** Susceptibility of Gram-negative rods to other antibiotics among strains that were susceptible and resistant to fosfomycin.

Pathogens	S/R	Susceptibility to Other Antibiotics (%)									
		Amikacin	Gentamicin	Imipenem	Meropenem	Ceftazidime	Cefepime	Piperacillin-Tazobactam	Ciprofloxacin	Levofloxacin	Tigecycline
*Enterobacterales*	Total	86	68.3	80	81.4	59.2	58.6	67	45.7	47.4	39.8
	S	91.4	74.4	86.2	87.3	66.4	65.8	74.6	52.3	54.1	47
	R	66.7	46.2	56.9	59.6	32.5	33.3	39.3	21.4	23.1	13.8
*Pseudomonas* spp.	Total	82.4	NT	63.6	73	71.2	NT	73.7	58.6	58.9	NT
	S	83.5	NT	66.4	75.5	71.9	NT	74.6	60.9	61.3	NT
	R	71.4	NT	35.7	46.2	64.3	NT	64.3	35.7	35.7	NT
*Acinetobacter* spp.	Total	5.7	4.6	5.7	5.9	NT	NT	4.6	2.3	2.5	NT

NT: not tested.

**Table 7:** Susceptibility of Gram-positive cocci to other antibiotics among strains that were susceptible and resistant to fosfomycin.

Pathogens	S/R	Susceptibility to Other Antibiotics (%)							
		Methicillin (MSS)	Gentamicin	Ceftaroline	Ciprofloxacin	Trimethoprim-Sulfamethoxazole	Vancomycin	Linezolid	Tigecycline
*S. aureus*	Total	61.5	96.1	98.9	49	100	100	100	NT
	S	62	97	98.9	49.5	100	100	100	NT
	R	n = 0	n = 0	n = 1	n = 0	n = 0	n = 1	n = 1	NT
CNS	Total	26	59.6	NT	50	66.1	98.2	92.7	NT
	S	34.9	67.4	NT	53.5	71.4	97.6	95	NT
	R	0	35.7	NT	38.5	50	100	85.7	NT
*Enterococcus* spp.	Total	NT	72	NT	NT	NT	76	98	100

NT: not tested, MSS: methicillin-susceptible *Staphylococcus*, CNS: coagulase-negative *Staphylococcus*.

## Data Availability

Not applicable.

## Supplementary Materials

The following supporting information can be downloaded at: https://www.mdpi.com/article/10.3390/pathogens11121441/s1. Figure S1: Reading the MIC values in the reference method; Figure S2: Reading the MIC values in the reference method for *Proteus* spp. strains. Table S1: *Klebsiella spp.* vs. *E. coli* Contingency Table.

## References

[1] Marino A., Stracquadani S., Bellanca C.M., Augello E., Ceccarelli M., Cantarella G., Bernardini R., Nunnari G., Cacopardo B. (2022). Oral Fosfomycin Formulation in Bacterial Prostatitis: New Role for an Old Molecule-Brief Literature Review and Clinical Considerations. Infect. Dis. Rep..

[2] Karaiskos I., Galani L., Sakka V., Gkoufa A., Sopilidis O., Chalikopoulos D., Alivizatos G., Giamarellou E. (2019). Oral fosfomycin for the treatment of chronic bacterial prostatitis. J. Antimicrob. Chemother..

[3] European Medicines Agency Assessment Report. EMA (Online). https://www.ema.europa.eu/en/documents/referral/fosfomycin-article-31-referralassessment-report_en.pdf.

[4] Zhanel G.G., Zhanel M.A., Karlowsky J.A. (2019). Intravenous Fosfomycin: An Assessment of Its Potential for Use in the Treatment of Systemic Infections in Canada. Can. J. Infect. Dis. Med. Microbiol..

[5] Kaneko M., Emoto Y., Emoto M. (2016). A Simple, Reproducible, Inexpensive, Yet Old-Fashioned Method for Determining Phagocytic and Bactericidal Activities of Macrophages. Yonsei Med. J..

[6] Meena V.D., Dotaniya M.L., Saha J.K., Patra A.K. (2015). Antibiotics and antibiotic resistant bacteria in wastewater: Impact on environment, soil microbial activity and human health. Afr. J. Microbiol. Res..

[7] Díez-Aguilar M., Cantón R. (2019). New microbiological aspects of fosfomycin. Rev. Esp. Quimioter..

[8] Dijkmans A.C., Zacarías N.V.O., Burggraaf J., Mouton J.W., Wilms E.B., van Nieuwkoop C., Touw D.J., Stevens J., Kamerling I.M.C. (2017). Fosfomycin: Pharmacological, Clinical and Future Perspectives. Antibiotics.

[9] Cao Y., Peng Q., Li S., Deng Z., Gao J. (2019). The intriguing biology and chemistry of fosfomycin: The only marketed phosphonate antibiotic. RSC Adv..

[10] Williams P.C.M. (2020). Potential of fosfomycin in treating multidrug-resistant infections in children. J. Paediatr. Child Health.

[11] AD Fosfomycin 0.25-256 Device for Fosfomycin Susceptibility Testing with the Agar Dilution Method. Liofilchem (Online). http://www.liofilchem.net/login/pd/ifu/77061_IFU.pdf.

[12] European Committee on Antimicrobial Susceptibility Testing Breakpoint Tables for Interpretation of MICs and Zone Diameters. Version 12.0. https://www.eucast.org/fileadmin/src/media/PDFs/EUCAST_files/Breakpoint_tables/v_12.0_Breakpoint_Tables.pdf.

[13] Van den Bijllaardt W., Schijffelen M.J., Bosboom R.W., Cohen Stuart J., Diederen B., Kampinga G., Muller A.E. (2018). Susceptibility of ESBL Escherichia coli and Klebsiella pneumoniae to fosfomycin in the Netherlands and comparison of several testing methods including Etest, MIC test strip, Vitek2, Phoenix and disc diffusion. J. Antimicrob. Chemother..

[14] The European Committee on Antimicrobial Susceptibility Testing (EUCAST) (2021). Routine and Extended Internal Quality Control for MIC Determination and Disk Diffusion as Recommended by EUCAST. Version 11.0. http://www.eucast.org.

[15] EUCAST General Consultation on Fosfomycin IV Breakpoints Consultation Period 14 May to (Extension) 15 July 2022. https://www.eucast.org/publications_and_documents/consultations/.

[16] CLSI (2019). Performance Standards for Antimicrobial Susceptibility Testing.

[17] Doyle D., Peirano G., Lascols C., Lloyd T., Church D.L., Pitout J.D. (2012). Laboratory detection of Enterobacteriaceae that produce carbapenemases. J. Clin. Microbiol..

[18] MacDonald L.V., Chibabhai V. (2019). Evaluation of the RESIST-4 O.K.N.V immunochromatographic lateral flow assay for the rapid detection of OXA-48, KPC, NDM and VIM carbapenemases from cultured isolates. Access Microbiol..

[19] (2000). European Committee for Antimicrobial Susceptibility Testing (EUCAST) of the European Society of Clinical Microbiology and Infectious Diseases (ESCMID), Determination of minimum inhibitory concentrations (MICs) of antibacterial agents by agar dilution. Clin. Microbiol. Infect..

[20] WHO Regional Office for Europe, European Centre for Disease Prevention and Control (2022). Antimicrobial Resistance Surveillance in Europe 2022–2020 Data.

[21] Data from the ECDC Surveillance Atlas Antimicrobial Resistance The Surveillance Atlas of Infectious Diseases. 2022–2020.2022 Data. https://atlas.ecdc.europa.eu/public/index.aspx?Dataset=27&HealthTopic=4.

[22] Elham B., Fawzia A. (2019). Colistin resistance in *Acinetobacter baumannii* isolated from critically ill patients: Clinical characteristics, antimicrobial susceptibility and outcome. Afr. Health Sci..

[23] Papathanakos G., Andrianopoulos I., Papathanasiou A., Priavali E., Koulenti D., Koulouras V. (2020). Colistin-Resistant *Acinetobacter Baumannii* Bacteremia: A Serious Threat for Critically Ill Patients. Microorganisms.

[24] Cafiso V., Stracquadanio S., Lo Verde F., Gabriele G., Mezzatesta M.L., Caio C., Pigola G., Ferro A., Stefani S. (2019). Colistin Resistant *A. baumannii*: Genomic and Transcriptomic Traits Acquired Under Colistin Therapy. Front. Microbiol..

[25] Malik S., Kaminski M., Landman D., Quale J. (2020). Cefiderocol Resistance in *Acinetobacter baumannii*: Roles of β-Lactamases, Siderophore Receptors, and Penicillin Binding Protein 3. Antimicrob. Agents Chemother..

[26] Smoke S.M., Brophy A., Reveron S., Iovleva A., Kline E.G., Marano M., Miller L.P., Shields R.K. (2022). Evolution and Transmission of Cefiderocol-Resistant *Acinetobacter baumannii* During an Outbreak in the Burn Intensive Care Unit. Clin. Infect. Dis..

[27] Karakonstantis S., Rousaki M., Kritsotakis E.I. (2022). Cefiderocol: Systematic Review of Mechanisms of Resistance, Heteroresistance and In Vivo Emergence of Resistance. Antibiotics.

[28] Putensen C., Ellger B., Sakka S.G., Weyland A., Schmidt K., Zoller M., Weiler N., Kindgen-Milles D., Jaschinski U., Weile J. (2019). Current clinical use of intravenous fosfomycin in ICU patients in two European countries. Infection.

[29] Zirpe K.G., Mehta Y., Pandit R., Pande R., Deshmukh A.M., Patil S., Bhagat S., Barkate H. (2021). A Real-world Study on Prescription Pattern of Fosfomycin in Critical Care Patients. Indian J. Crit. Care Med..

[30] Pontikis K., Karaiskos I., Bastani S., Dimopoulos G., Kalogirou M., Katsiari M., Oikonomou A., Poulakou G., Roilides E., Giamarellou H. (2014). Outcomes of critically ill intensive care unit patients treated with fosfomycin for infections due to pandrug-resistant and extensively drug-resistant carbapenemase-producing Gram-negative bacteria. Int. J. Antimicrob. Agents.

[31] Tsegka K.G., Voulgaris G.L., Kyriakidou M., Falagas M.E. (2020). Intravenous fosfomycin for the treatment of patients with central nervous system infections: Evaluation of the published evidence. Expert Rev. Anti Infect. Ther..

[32] Tsegka K.G., Voulgaris G.L., Kyriakidou M., Kapaskelis A., Falagas M.E. (2022). Intravenous fosfomycin for the treatment of patients with bone and joint infections: A review. Expert Rev. Anti Infect. Ther..

[33] Stracquadanio S., Musso N., Costantino A., Lazzaro L.M., Stefani S., Bongiorno D. (2021). *Staphylococcus aureus* Internalization in Osteoblast Cells: Mechanisms, Interactions and Biochemical Processes. What Did We Learn from Experimental Models?. Pathogens.

[34] Zelmer A.R., Nelson R., Richter K., Atkins G.J. (2022). Can intracellular *Staphylococcus aureus* in osteomyelitis be treated using current antibiotics? A systematic review and narrative synthesis. Bone Res..

[35] Valour F., Trouillet-Assant S., Riffard N., Tasse J., Flammier S., Rasigade J.P., Chidiac C., Vandenesch F., Ferry T., Laurent F. (2015). Antimicrobial activity against intraosteoblastic *Staphylococcus aureus*. Antimicrob. Agents Chemother..

[36] Morata L., Soriano A. (2019). The role of fosfomycin in osteoarticular infection. Rev. Esp. Quimioter..

[37] Li G., Standing J.F., Bielicki J., Hope W., van den Anker J., Heath P.T., Sharland M. (2017). The Potential Role of Fosfomycin in Neonatal Sepsis Caused by Multidrug-Resistant Bacteria. Drugs.

[38] Baquero F., Hortelano J.G., Navarro M., Scarpellini A., Jara P., Cañedo T., Rodríguez A. (1977). Antibiotherapy of *Serratia marcescens* septicemia in children. Chemotherapy.

[39] Corti N., Sennhauser F.H., Stauffer U.G., Nadal D. (2003). Fosfomycin for the initial treatment of acute haematogenous osteomyelitis. Arch. Dis. Child..

[40] Li Y., Zheng B., Li Y., Zhu S., Xue F., Liu J. (2015). Antimicrobial Susceptibility and Molecular Mechanisms of Fosfomycin Resistance in Clinical *Escherichia coli* Isolates in Mainland China. PLoS ONE.

[41] Banerjee S., Sengupta M., Sarker T.K. (2017). Fosfomycin susceptibility among multidrug-resistant, extended-spectrum beta-lactamase-producing, carbapenem-resistant uropathogens. Indian J. Urol..

[42] Fajfr M., Louda M., Paterová P., Ryšková L., Pacovský J., Košina J., Žemličková H., Broďák M. (2017). The susceptibility to fosfomycin of Gram-negative bacteria isolates from urinary tract infection in the Czech Republic: Data from a unicentric study. BMC Urol..

[43] Parisio E.M., Camarlinghi G., Coppi M., Niccolai C., Antonelli A., Nardone M., Vettori C., Giani T., Mattei R., Rossolini G.M. (2021). Evaluation of commercial AD Fosfomycin test for susceptibility testing of multidrug-resistant Enterobacterales and *Pseudomonas aeruginosa*. Clin. Microbiol. Infect..

[44] Xu W., Chen T., Wang H., Zeng W., Wu Q., Yu K., Xu Y., Zhang X., Zhou T. (2020). Molecular Mechanisms and Epidemiology of Fosfomycin Resistance in *Staphylococcus aureus* Isolated from Patients at a Teaching Hospital in China. Front. Microbiol..

[45] Souza R.B., Trevisol D.J., Schuelter-Trevisol F. (2015). Bacterial sensitivity to fosfomycin in pregnant women with urinary infection. Braz. J. Infect. Dis..

[46] Falagas M.E., Roussos N., Gkegkes I.D., Rafailidis P.I., Karageorgopoulos D.E. (2009). Fosfomycin for the treatment of infections caused by Gram-positive cocci with advanced antimicrobial drug resistance: A review of microbiological, animal and clinical studies. Expert Opin. Investig. Drugs.

[47] Perez Fernandez P., Herrera I., Martinez P., Gómez-Lus M.L., Prieto J. (1995). Enhancement of the susceptibility of *Staphylococcus aureus* to phagocytosis after treatment with fosfomycin compared with other antimicrobial agents. Chemotherapy.

[48] Trautmann M., Meincke C., Vogt K., Ruhnke M., Lajous-Petter A.M. (1992). Intracellular bactericidal activity of fosfomycin against staphylococci: A comparison with other antibiotics. Infection.

[49] Krause R., Patruta S., Daxbock F., Fladere P., Wenisch C. (2001). The effect of fosfomycin on neutrophil function. J. Antimicrob. Chemother..

[50] Tullio V., Cuffini A.M., Banche G., Mandras N., Allizond V., Roana J., Giacchino F., Bonello F., Ungheri D., Carlone N.A. (2008). Role of fosfomycin tromethamine in modulating non-specific defence mechanisms in chronic uremic patients towards ESBL-producing *Escherichia coli*. Int. J. Immunopathol. Pharmacol..

[51] Hamada M., Honda J., Yoshimuta T., Fumimori T., Okamoto M., Aizawa H. (2002). Fosfomycin inhibits neutrophil function via a protein kinase C-dependent signaling pathway. Int. Immunopharmacol..

[52] Castaneda-García A., Rodríguez-Rojas A., Guelfo J.R., Blazquez J. (2009). The Glycerol-3-Phosphate Permease GlpT Is the Only Fosfomycin Transporter in *Pseudomonas aeruginosa*. J. Bacteriol..

[53] European Committee on Antimicrobial Susceptibility Testing MIC and Zone Diameter Distributions and ECOFFs. https://www.eucast.org/mic_distributions_and_ecoffs/.

[54] Mirakhur A., Gallagher M.J., Ledson M.J., Hart C.A., Walshaw M.J. (2003). Fosfomycin therapy for multiresistant *Pseudomonas aeruginosa* in cystic fibrosis. J. Cyst. Fibros..

[55] Paul M., Carrara E., Retamar P., Tängdén T., Bitterman R., Bonomo R.A., de Waele J., Daikos G.L., Akova M., Harbarth S. (2022). European Society of Clinical Microbiology and Infectious Diseases (ESCMID) guidelines for the treatment of infections caused by multidrug-resistant Gram-negative bacilli (endorsed by European society of intensive care medicine). Clin. Microbiol. Infect..

[56] Papp-Wallace K.M., Zeiser E.T., Becka S.A., Park S., Wilson B.M., Winkler M.L., D’Souza R., Singh I., Sutton G., Fouts D.E. (2019). Ceftazidime-Avibactam in Combination with Fosfomycin: A Novel Therapeutic Strategy Against Multidrug-Resistant *Pseudomonas aeruginosa*. J. Infect. Dis..

[57] Di X., Wang R., Liu B., Zhang X., Ni W., Wang J., Liang B., Cai Y., Liu Y. (2015). In vitro activity of fosfomycin in combination with colistin against clinical isolates of carbapenem-resistant *Pseudomas aeruginosa*. J. Antibiot..

[58] Albiero J., Mazucheli J., Barros J.P.D.R., Szczerepa M.M.D.A., Nishiyama S.A.B., CarraraMarroni F.E., Sy S., Fidler M., Sy S.K.B., Tognim M.C.B. (2019). Pharmacodynamic attainment of the synergism of meropenem and fosfomycin combination against *Pseudomonas aeruginosa* producing metallo-β-lactamase. Antimicrob. Agents Chemother..

[59] Walsh C.C., McIntosh M.P., Peleg A.Y., Kirkpatrick C.M., Bergen P.J. (2015). In vitro pharmacodynamics of fosfomycin against clinical isolates of *Pseudomonas aeruginosa*. J. Antimicrob. Chemother..

[60] Williams P.C.M., Waichungo J., Gordon N.C., Sharland M., Murunga S., Kamau A., Berkley J.A. (2019). The potential of fosfomycin for multi-drug resistant sepsis: An analysis of in vitro activity against invasive paediatric Gram-negative bacteria. J. Med. Microbiol..

[61] Díez-Aguilar M., Morosini M.I., del Campo R., García-Castillo M., Zamora J., Cantón R. (2013). In vitro activity of fosfomycin against a collection of clinical *Pseudomonas aeruginosa* isolates from 16 Spanish hospitals: Establishing the validity of standard broth microdilution as susceptibility testing method. Antimicrob. Agents Chemother..

[62] Gopichand P., Agarwal G., Natarajan M., Mandal J., Deepanjali S., Parameswaran S., Dorairajan L.N. (2019). In vitro effect of fosfomycin on multi-drug resistant gram-negative bacteria causing urinary tract infections. Infect. Drug Resist..

[63] Effah C.Y., Sun T., Liu S., Wu Y. (2020). Klebsiella pneumoniae: An increasing threat to public health. Ann. Clin. Microbiol. Antimicrob..

[64] Lan P., Jiang Y., Zhou J., Yu Y. (2021). A global perspective on the convergence of hypervirulence and carbapenem resistance in *Klebsiella pneumoniae*. J. Glob. Antimicrob. Resist..

[65] Hawkey P.M., Warren R.E., Livermore D.M., McNulty C.A.M., Enoch D.A., Otter J.A., Wilson A.P.R. (2018). Treatment of infections caused by multidrug-resistant Gram-negative bacteria: Report of the British Society for Antimicrobial Chemotherapy/Healthcare Infection Society/British Infection Association Joint Working Party. J. Antimicrob. Chemother..

[66] Mączyńska B., Paleczny J., Oleksy-Wawrzyniak M., Choroszy-Król I., Bartoszewicz M. (2021). In Vitro Susceptibility of Multi-Drug Resistant *Klebsiella pneumoniae* Strains Causing Nosocomial Infections to Fosfomycin. A Comparison of Determination Methods. Pathogens.

[67] Demirci-Duarte S., Unalan-Altintop T., Gulay Z., Sari Kaygisiz A.N., Cakar A., Gur D. (2020). In vitro susceptibility of OXA-48, NDM, VIM and IMP enzyme- producing *Klebsiella* spp. and *Escherichia coli* to Fosfomycin. J. Infect. Dev. Ctries..

[68] Flamm R.K., Rhomberg P.R., Watters A.A., Sweeney K., Ellis-Grosse E.J., Shortridge D. (2019). Activity of fosfomycin when tested against US contemporary bacterial isolates. Diagn. Microbiol. Infect. Dis..

[69] Grabein B., Graninger W., Rodríguez Bańo J., Dinh A., Liesenfeld D.B. (2017). Intravenous fosfomycin—Back to the future. Systematic review and meta-analysis of the clinical literature. Clin. Microbiol. Infect..

[70] Zhao M., Bulman Z.P., Lenhard J.R., Satlin M.J., Kreiswirth B.N., Walsh T.J., Marrocco A., Bergen P.J., Nation R.L., Li J. (2017). Pharmacodynamics of colistin and fosfomycin: A ‘treasure trove’ combination combats KPC-producing Klebsiella pneumoniae. J. Antimicrob. Chemother..

[71] Perdigão Neto L.V., Oliveira M.S., Martins R.C.R., Marchi A.P., Gaudereto J.J., da Costa L.A.T.J., de Lima L.F.A., Takeda C.F.V., Costa S.F., Levin A.S. (2019). Fosfomycin in severe infections due to genetically distinct pan-drug-resistant Gram-negative microorganisms: Synergy with meropenem. J. Antimicrob. Chemother..

[72] Folgori L., Ellis S.J., Bielicki J.A., Heath P.T., Sharland M., Balasegaram M. (2017). Tackling antimicrobial resistance in neonatal sepsis. Lancet Glob. Health.

[73] Rieg S., Ernst A., Peyerl-Hoffmann G., Joost I., Camp J., Hellmich M., Kern W.V., Kaasch A.J., Seifert H. (2020). Combination therapy with rifampicin or fosfomycin in patients with Staphylococcus aureus bloodstream infection at high risk for complications or relapse: Results of a large prospective observational cohort. J. Antimicrob. Chemother..

[74] AL-Quraini M., Rizvi M., AL-Jabri Z., Sami H., AL-Muzahmi M., AL-Muharrmi Z., Taneja N., AL-Busaidi I., Soman R. (2022). Assessment of In-Vitro Synergy of Fosfomycin with Meropenem, Amikacin and Tigecycline in Whole Genome Sequenced Extended and Pan Drug Resistant Klebsiella Pneumoniae: Exploring A Colistin Sparing Protocol. Antibiotics.

[75] Singkham-in U., Chatsuwan T. (2022). Synergism of imipenem with fosfomycin associated with the active cell wall recycling and heteroresistance in *Acinetobacter calcoaceticus*-*baumannii* complex. Sci. Rep..

[76] Ruiz Ramos J., Salavert Lletí M. (2019). Fosfomycin in infections caused by multidrug-resistant Gram-negative pathogens. Rev. Esp. Quimioter..

[77] Yusuf E., Bax H.I., Verkaik N.J., van Westreenen M. (2021). An Update on Eight “New” Antibiotics against Multidrug-Resistant Gram-Negative Bacteria. J. Clin. Med..

[78] Nwabor O.F., Terbtothakun P., Voravuthikunchai S.P., Chusri S. (2021). Evaluation of the Synergistic Antibacterial Effects of Fosfomycin in Combination with Selected Antibiotics against Carbapenem-Resistant *Acinetobacter baumannii*. Pharmaceuticals.

[79] Bassetti M., Garau J. (2021). Current and future perspectives in the treatment of multidrug-resistant Gram-negative infections. J. Antimicrob. Chemother..

[80] Abbott I.J., van Gorp E., van der Meijden A., Wijma R.A., Meletiadis J., Roberts J.A., Mouton J.W., Peleg A.Y. (2020). Oral Fosfomycin Treatment for Enterococcal Urinary Tract Infections in a Dynamic *In Vitro* Model. Antimicrob. Agents Chemother..

[81] Guo Y., Tomich A.D., McElheny C.L., Cooper V.S., Tait-Kamradt A., Wang M., Hu F., Rice L.B., Sluis-Cremer N., Doi Y. (2017). High-Level Fosfomycin Resistance in Vancomycin-Resistant Enterococcus faecium. Emerg. Infect. Dis..

[82] Vardakas K.Z., Legakis N.J., Triarides N., Falagas M.E. (2016). Susceptibility of contemporary isolates to fosfomycin: A systematic review of the literature. Int. J. Antimicrob. Agents.

