# Supplementary material for: Assessment of the Susceptibility of Clinical Gram-Negative and Gram-Positive Bacterial Strains to Fosfomycin and Significance of This Antibiotic in Infection Treatment
Source: Pathogens. 2022 Nov 30;11(12):1441. doi: 10.3390/pathogens11121441 (PMC9786176; doi:10.3390/pathogens11121441)
Supplement: Supplementary file 1 [file pathogens-11-01441-s001.zip › pathogens-1991336-supplementary.pdf]

pathogens

Supplementary materials

Susceptibility assessment of clinical gram-negative and gram-positive bacterial strains to fosfomycin and significance of this antibiotic in infection treatment

Beata Kowalska-Krochmal[1*], Beata Mączyńska[1], Danuta Rurańska- Smutnicka[1], Anna Secewicz[1], Grzegorz Krochmal, Małgorzata Bartelak[1], Aleksandra Górzyńska[1], Klaudyna Laufer[2], Krystyna Woronowicz[3], Joanna Łubniewska[4], Jolanta Łappo[5], Magdalena Czwartos[6], Ruth Dudek-Wicher[1]

1 Department of Pharmaceutical Microbiology and Parasitology, Faculty of Pharmacy, Medical University of Silesian Piasts in Wroclaw, Borowska 211, 50-556 Wroclaw, Poland
2 Laboratory Diagnostics Department, Jan Mikulicz-Radecki University Teaching Hospital Borowska 213, 50-556 Wrocław, Poland
3 Synevo, T. Marciniak Lower Silesian Specialized Hospital, Gen. A. E. Fieldorfa 2, 54-049 Wrocław
4 Microbiological laboratory, Provincial Specialized Hospital in Legnica, Iwaszkiewicza 5, 59-220 Legnica
5 Microbiological laboratory, A. Sokolowski Specialized Hospital in Walbrzych, Health dept. in Klodzko; A. Sokołowskiego 4, 58-309 Walbrzych
6 Bacteriology Laboratory, Provincial Specialized Hospital in Klodzko, Szpitalna 1A, 57-300 Klodzko
* Correspondence: : beakk103@gmail.com

Citation: Kowalska-Krochmal, B.; Mączyńska, B.; Rurańska-Smutnicka, D.; Secewicz, A.; Krochmal, G.; Bartelak, M.; Górzyńska, A.; Laufer, K.; Woronowicz, K.; Łubniewska, J.; et al. Assessment of the Susceptibility of Clinical Gram-Negative and Gram-Positive Bacterial Strains to Fosfomycin and Significance of This Antibiotic in Infection Treatment. *Pathogens* **2022**, *11*, 1441. https://doi.org/10.3390/pathogens11121441

Academic Editors: Andaleeb Sajid and Gunjan Arora

Received: 10 October 2022
Accepted: 29 November 2022
Published: 30 November 2022

Publisher's Note: MDPI stays neutral with regard to jurisdictional claims in published maps and institutional affiliations.

Copyright: © 2022 by the authors. Submitted for possible open access publication under the terms and conditions of the Creative Commons Attribution (CC BY) license (https://creativecommons.org/licenses/by/4.0/).

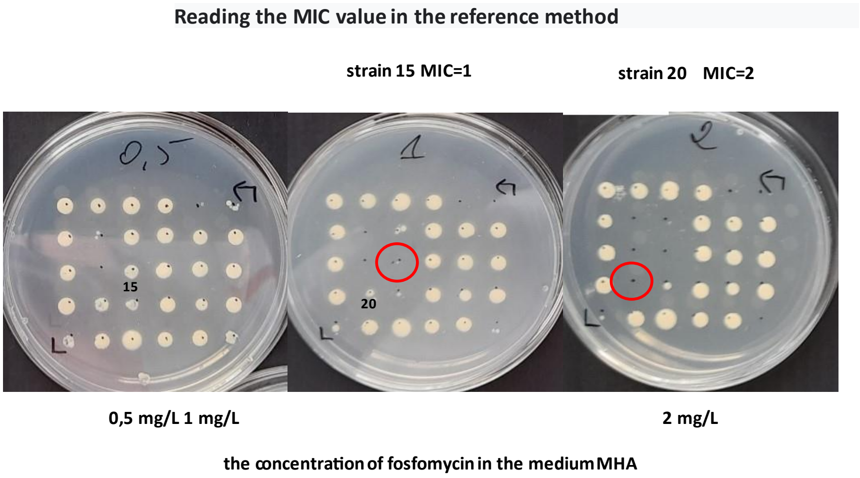

Figure S1. Reading the MIC value in the reference method

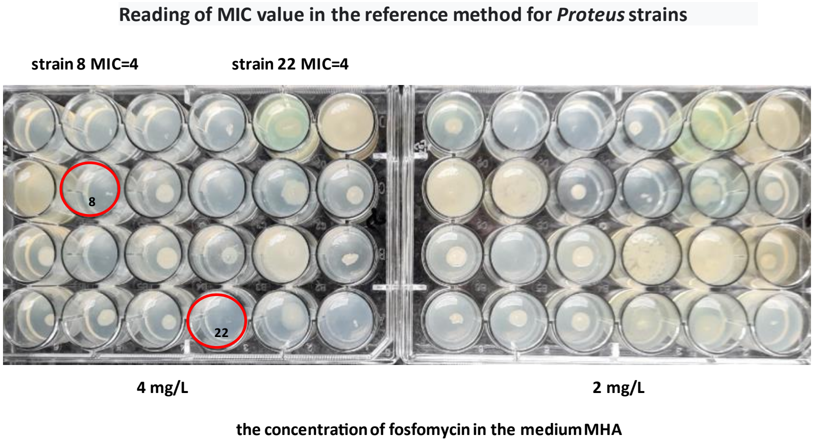

Figure S2. Reading the MIC value in the reference method for *Proteus* spp. strains

1. Statistical analysis explanation

1.1 Susceptibility Testing

Statistical tests were performed using Scipy[1] library in Python programming language. The differences in susceptibility between the groups (Strains, Materials, Resistance Mechanism) were compared with *Chi-square test of independence of variables*[2]. The test results were considered significant for ***p-value*** lower than 0.05.

1.2 Methodology Explanation

For all of the groups compared (Strains, Materials, Resistance Mechanism) the same methodology was followed. Contingency tables were created for each relevant pair of the groups mentioned. An example contingency table[3] for *Klebsiella spp.* vs *E.coli* is shown in **Table S1.** Based on contingency tables, ^{2} *(Chi-square)* statistic was calculated.

Table S1. *Klebsiella spp. vs E.coli* Contingency Table

	S	R
**Klebsiella** spp.	165	85
**E.coli**	170	11

1.3 Results Explained

For each of the groups analyzed (Strains, Materials, Resistance Mechanism), for each pair tested (Strain 1 vs Strain 2, Material 1 vs Material 2 etc.) the same Null Hypothesis can be formulated.

H_0

Proportion of susceptible observations is independent of the Strain/Material/Mechanism type (on a pair level)

If p-value obtained is lower than our significance level = 0.05, then we reject the Null Hypothesis. The Alternative Hypothesis is as follows.

H_1

Proportion of susceptible observations is different for different type of Strain/Material/Mechanism (on a pair level)

This means, for the pairs tested where our ***p-value*** is lower than 0.05, we might not rule out that there is significant difference in susceptibility between them.

---

[1] https://scipy.org/citing-scipy/

[2] https://docs.scipy.org/doc/scipy/reference/generated/scipy.stats.chi2_contingency.html

[3] https://sphweb.bumc.bu.edu/otlt/mph-modules/bs/bs704_hypothesistesting-chisquare/bs704_hypothesistesting-chisquare_print.html
